# Phage-Antibiotic Synergy Is Driven by a Unique Combination of Antibacterial Mechanism of Action and Stoichiometry

**DOI:** 10.1128/mBio.01462-20

**Published:** 2020-08-04

**Authors:** Carmen Gu Liu, Sabrina I. Green, Lorna Min, Justin R. Clark, Keiko C. Salazar, Austen L. Terwilliger, Heidi B. Kaplan, Barbara W. Trautner, Robert F. Ramig, Anthony W. Maresso

**Affiliations:** a Tailored Antibacterials and Innovative Laboratories for Phage (Φ) Research (TAILΦR), Department of Molecular Virology and Microbiology, Baylor College of Medicine, Houston, Texas, USA; b School of Medicine, Baylor College of Medicine, Houston, Texas, USA; c Department of Microbiology and Molecular Genetics, University of Texas Health Science Center at Houston, Houston, Texas, USA; d Michael E. DeBakey Veterans Affairs Medical Center, Department of Medicine, Baylor College of Medicine, Houston, Texas, USA; University of Texas Southwestern Medical Center Dallas

**Keywords:** synogram, synography, phage, antibiotic, adjuvant, synergy, phage therapy, *Escherichia coli*, bacteriophage, clinical isolate, combinatorial treatment

## Abstract

The continued rise in antibiotic resistance is precipitating a medical crisis. Bacteriophage (phage) has been hailed as one possible therapeutic option to augment the efficacy of antibiotics. However, only a few studies have addressed the synergistic relationship between phage and antibiotics. Here, we report a comprehensive analysis of phage-antibiotic interaction that evaluates synergism, additivism, and antagonism for all classes of antibiotics across clinically achievable stoichiometries. We combined an optically based real-time microtiter plate readout with a matrix-like heat map of treatment potencies to measure phage and antibiotic synergy (PAS), a process we term synography. Phage-antibiotic synography was performed against a pandemic drug-resistant clonal group of extraintestinal pathogenic Escherichia coli (ExPEC) with antibiotic levels blanketing the MIC across seven orders of viral titers. Our results suggest that, under certain conditions, phages provide an adjuvating effect by lowering the MIC for drug-resistant strains. Furthermore, synergistic and antagonistic interactions are highly dependent on the mechanism of bacterial inhibition by the class of antibiotic paired to the phage, and when synergism is observed, it suppresses the emergence of resistant cells. Host conditions that simulate the infection environment, including serum and urine, suppress PAS in a bacterial growth-dependent manner. Lastly, two different related phages that differed in their burst sizes produced drastically different synograms. Collectively, these data suggest lytic phages can resuscitate an ineffective antibiotic for previously resistant bacteria while also synergizing with antibiotics in a class-dependent manner, processes that may be dampened by lower bacterial growth rates found in host environments.

## Abstract (precis)

Bacteriophage (phage) therapy is a promising approach to combat the rise of multidrug-resistant bacteria. Currently, the preferred clinical modality is to pair phage with an antibiotic, a practice thought to improve efficacy. However, antagonism between phage and antibiotics has been reported, the choice of phage and antibiotic is not often empirically determined, and the effect of the host factors on the effectiveness is unknown. Here, we interrogate phage-antibiotic interactions across antibiotics with different mechanisms of action. Our results suggest that phage can lower the working MIC for bacterial strains already resistant to the antibiotic, is dependent on the antibiotic class and stoichiometry of the pairing, and is dramatically influenced by the host microenvironment.

## INTRODUCTION

A major public health crisis is the alarming increase of infections caused by antibiotic-resistant bacteria, which are responsible for approximately 2.8 million infections in the United States alone (1). In 2014, the World Health Organization (WHO) reported that a post-antibiotic era, in which antibiotics are largely ineffective, is a possible fate for the 21st century (2). The post-antibiotic era is described as a period where antibiotics fail to target multidrug-resistant bacteria. Five years later in 2019, the Centers for Disease Control and Prevention (CDC) in its Antibiotic Resistance Threat Report claimed that the post-antibiotic era had already arrived (1). In a report released by a U.K. Commission, it was concluded that 10 million deaths a year, at a cost of around 3 trillion dollars, will occur due to drug-resistant infections by the year 2050 (3). Furthermore, the exchange of genetic elements that confer resistance is common among members of the human microbiome, resistance has developed against every major chemical class of antibiotics, and the overuse of antibiotics in patients and agriculture selects for such strains.

Of great concern is the pandemic clonal group called extraintestinal pathogenic Escherichia coli (ExPEC) with the sequence type 131 (ST131) (4). This group demonstrates a highly virulent phenotype and is a prominent cause of urinary tract, peritoneal, bloodstream, and neonatal meningitis infections while also being resistant to fluoroquinolone and β-lactam antibiotics (5, 6). In addition, ExPEC is a highly versatile pathogen composed of many additional circulating sequence types possessing a plethora of virulence and resistance genes (4).

To address this growing problem, several alternatives to traditional chemical antibiotics have been explored: antibody therapy, antimicrobial peptides, probiotics, metal chelation, and even incentives to expedite the drug-approval process and stimulate new antibiotic development (7). Although promising, all of these approaches are limited by the fact that the antimicrobial agent cannot change or adapt in real time. That is, should resistance arise, the agent has little ability to become effective again, especially considering the significant investment in time and dollars needed to bring new drugs to the market. The ability of bacteria to mutate quickly and to evolve around such approaches are both their greatest asset and a biological reality that disincentivizes investment in the antibiotic-making business. In contrast, bacteriophages, viruses that infect and kill bacteria, are as equally evolvable and adaptable as bacteria, in addition to being the most numerous replicating entity on Earth (estimated to be around 10^31^ total particles) (8). The adaptability and sheer number of phages imply that they are the largest repository of antibacterial information available to modern medicine. Furthermore, phages have been used to treat bacterial infections for decades in Eastern Europe, and recent compassionate care cases in the United States and United Kingdom have demonstrated clinical success (9, 10). Phages are also specific for a given bacterial species (even strain), meaning off-target killing of “good” bacteria in our microbiome can be minimized. Phages also amplify at the site of the infection and therefore self-dose, clearing when no longer needed due to excretion or breakdown in the host (11). Finally, phages are generally regarded as safe by the Food and Drug Administration (FDA), with millions of phage particles ingested every day in our food and water (12). These characteristics allow phages to become a potentially better alternative than chemical compounds for therapy, which, if developed through the normal pharma pipeline, may take 10 years and a billion dollars to bring to market.

One of the more attractive and feasible use of phages is to combine them with clinically used antibiotics (13). The combined use of phage and antibiotics may result in a number of outcomes. The two agents may act additively, that is, the sum of their individual effects is equal to their combinatorial efficacy. They may also act synergistically; their total efficacy is much greater than each individual action. A third result is no effect, owing to the lack of action of each individual agent. Finally, there may be antagonism whereby the molecular action of one of the agents somehow interferes with the action of the other. In reported cases of phage therapy in the United States, the choice of antibiotic was often made on the bases of antibiogram data and the medical condition of the patient (14). However, the recent awareness of antagonism has propelled some *in vitro* assessments of phage-antibiotic action prior to treatment to select for synergistic combinations, a personalized approach that has led to satisfactory therapeutic outcomes in those cases (15, 16). Several phage-antibiotic combinations have been investigated *in vitro* and *in vivo* in multiple bacterial species (17, 18), but there have been mixed results with combinatorial treatment (13, 19, 20). For instance, quinolones can be synergistic with phages against Pseudomonas aeruginosa in one study while antagonistic in another (21, 22). Sometimes, there are even two types of interactions found with the same antibiotic when they are combined with phages (23). Moreover, phage-antibiotic synergy (PAS) is usually studied with only one or two concentrations of the antimicrobials, which are wholly insufficient in predicting combinatorial concentrations that are efficacious during treatment. For these reasons, we assessed the effect of lytic phage on bacterial killing as a function of the presence of a resistance gene, the mechanism of action of the antibiotic, the likelihood of resistance, and the influence of host environments on effectiveness of PAS. We did these studies with a characterized myovirus (ΦHP3) that targets a pandemic clonal group of highly virulent ExPEC (strain JJ2528). Our investigation suggests that for phage-antibiotic combination therapy, clinicians should consider (i) pairing antibiotics with phages whose production machinery within the bacterial cell does not rely on the bacterial process that are inhibited by the very antibiotics they wish to use, (ii) the stoichiometry of the interactions, and (iii) how the host environment may affect treatment efficacy.

(This article was submitted to an online preprint archive [24].)

## RESULTS

### Formation of a comprehensive antibiotic-phage synergy system—the synogram.

**FIG 1:**
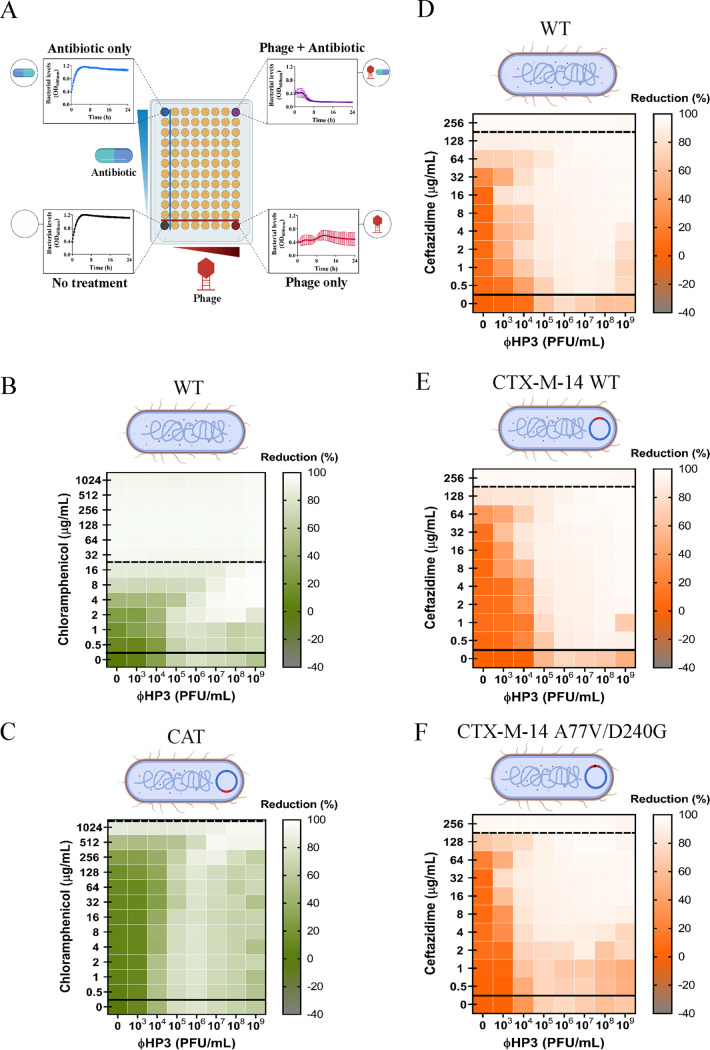
Effect of the bacterial resistance on phage-antibiotic synergy. A 100-fold diluted subculture of JJ2528 was incubated for 4 h, centrifuged, washed, adjusted to an OD_600_ of 1, and inoculated in a 96-well plate to which different treatments had been added to each well: phage alone (ΦHP3), antibiotic alone, phage-antibiotic combined, and untreated control. The OD_600_ was measured every 15 min for a total of 24 h at 37°C with shaking. (A) Synogram showing different treatments. The effects of antibiotic resistance on the gene and allele levels are shown as follow: chloramphenicol-ΦHP3 combined treatment on chloramphenicol sensitive wild-type JJ2528 (B) and chloramphenicol-resistant JJ2528 with CAT (chloramphenicol acetyltransferase) (C); ceftazidime-ΦHP3 combined treatment on wild-type JJ2528 (D), JJ2528 with β-lactamase CTX-M-14 wild-type that confers resistance against ceftazidime (E), and JJ2528 with β-lactamase CTX-M-14 A77V/D240G that confers increased resistance toward ceftazidime (F). Synograms (*t* = 24 h) represent the mean reduction percentage of each treatment from three biological replicates: Reduction (%) = [(OD_growthcontrol_ − OD_treatment_)/OD_growthcontrol_] × 100. The regions above the dashed lines indicate antibiotic-mediated killing with highly effective doses; the regions between the solid and dashed lines represent the interacting regions of the phage and antibiotic, and the regions below the solid lines indicate phage-mediated killing with ineffective antibiotic concentrations.

To understand the range of possible outcomes on bacterial growth when exposed to both phage and antibiotic, we assessed bacterial growth when exposed to concentrations of antibiotics that blanket the MIC across multiple orders of magnitude of phage titer over time in an optically based microtiter plate assay system. We call this synography, which is the process of determining antibiotic-phage effectiveness across many stoichiometries of each in clinically relevant situations. The primary phage used throughout the investigation, ΦHP3, is a highly effective killer of the ST131 ExPEC clinical isolates and is commonly used as a prototype in our laboratory (25). We have other characterized E. coli phages in our library that targeted JJ2528, but ΦHP3 was shown to have very good killing against JJ2528 both *in vitro* and in a mouse model of bacteremia (25, 26). Compared to other phages in the library, ΦHP3 can adsorb onto bacterial cells very quickly *in vitro*, had a decent burst size (60 PFU/cell) *in vitro*, can circulate to major organ tissues (liver, lung, spleen, and kidney) in mice, and was able to reduce disease severity in mice for the bacteremia model (25, 26). Since an inoculum effect was observed with this isolate in the presence of different antibiotics (see Fig. S2 in the supplemental material), bacteria were seeded at high inoculum to allow possible interaction between phage and antibiotic to occur under subinhibitory conditions. The optical density of the culture was then monitored at 37°C for 24 h. The absorbance was read as a stand-alone parameter and converted to a heat map that represents the percentage of reduction of the bacterial population, what we refer to as a synogram (Fig. 1A and see Fig. S5, raw data).

Throughout the study, we found that synograms seemed to follow a pattern specific to the antibiotic being tested and were generally divided into three sections: (i) an antibiotic-dominated-killing region, usually the upper division of the synograms, where antibiotics are effective and thus the killing pattern of the combination therapy closely tracks that of the antibiotic-only treated cells (Fig. 1A, top left); (ii) an interacting region, the middle section of the synograms, where the effect is a combination of both phage and antibiotic (additive, synergistic, or antagonistic) (Fig. 1A, top right); and lastly, (iii) a phage-dominated-killing region, the lower segment of the synograms, where antibiotics are ineffective and the killing activity is influenced more by the phage. Representing the data as a synogram achieves two main objectives that would not be realized by scanning the plethora of information generated from such data sets. First, it allows for a convenient colorimetric visualization of the effectiveness of phage-antibiotic interactions as their concentrations change relative to each other. By simply looking for points of low intensity, it allows one to determine the optimal concentration of each agent for maximal killing. Second, the synograms allow for an easy comparison of the global effectiveness of any antibiotic-phage combination across multiple agents and conditions. This includes an assessment of different classes of antibiotics, different phage sequence types, and different conditions, including those designed to simulate the host. One useful quantitative parameter when determining a phage-antibiotic concentration to use is the pairing of the two that reduces bacterial density by 90% or greater, a value that is denoted as the Synogram10 (Sn_10_). This way, multiples of the Sn_10_ (2×, 5×, etc.) can be used as a practical parameter when defining the concentration of each for an assay, experiment, or treatment.

### Effect of bacterial antibiotic resistance on combined phage-antibiotic efficacy.

Using the synogram as a proxy for combinatorial efficacy, we first asked how PAS may change when the only difference in the system is the absence or presence of an antibiotic resistance gene. For this, we assessed the phage-antibiotic killing dynamics of ExPEC strain JJ2528 lacking or containing the gene encoding the enzyme chloramphenicol acetyltransferase (CAT). CAT functions to transfer an acetyl group from coenzyme A to chloramphenicol, a modification that prevents the antibiotic from binding to the bacterial ribosome, prohibiting the inhibition of protein synthesis. We first examined the effect of each agent alone and in combination on wild-type JJ2528 that lacks the *cat* gene. The synogram of wild-type JJ2528 showed almost complete reduction (>95%) when bacterial cells were treated with ≥32 μg/ml of chloramphenicol, a region that is predominantly affected by the action of the antibiotic (Fig. 1B, above dashed line). In the ΦHP3-alone-treated cells, there was a progressive reduction from 10^3^ to 10^6^ PFU/ml. At higher phage titers, bacterial cells seemed to increase as phage titer increased (Fig. 1B, below solid line). This equates to a second growth of bacterial cells, likely resistance to the phage (examined below), a phenomenon that was observed throughout the study. Under combinatorial treatment, there is a nearly complete reduction of wild-type JJ2528 with concentrations as low as 1 μg/ml of chloramphenicol, suggesting that the addition of phage reduced the effective MIC of chloramphenicol 32-fold. The antibiotic, however, did not seem to appreciably reduce the phage titer needed for effective killing.

**FIG 2:**
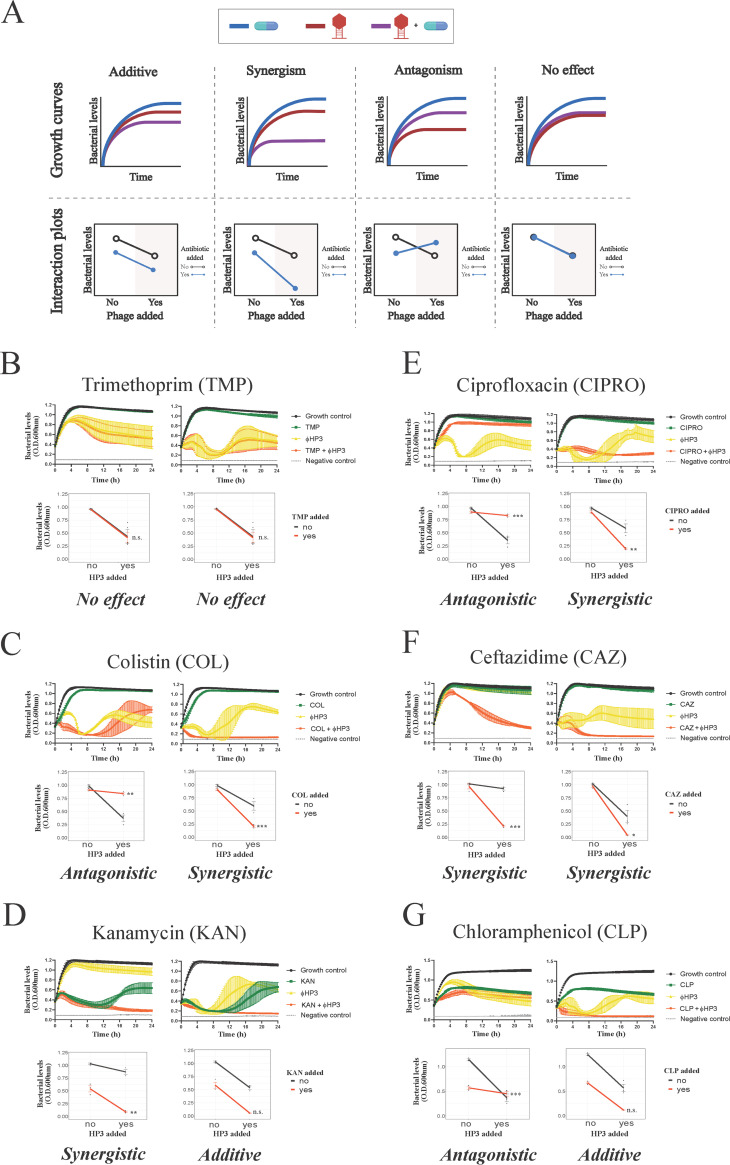
Growth characteristics and interaction plots for phage-antibiotic synergy. Bacterial growth over time was assessed for 24 h in the presence or absence of phage and antibiotic (top), and synergy was assessed via interaction plots (bottom). (A) Combination of phage and antibiotic resulted in additive, synergism, antagonism, and/or no effect. Representative interactions between ΦHP3 and antibiotics on wild-type JJ2528 (antibiotic dose plus phage titer): (B) trimethoprim, 0.5 μg/ml plus 10^5^ PFU/ml and 64 μg/ml plus 10^9^ PFU/ml; (C) colistin, 4 μg/ml plus 10^8^ PFU/ml and 4 μg/ml plus 10^9^ PFU/ml; (D) kanamycin, 16 μg/ml plus 10^4^ PFU/ml and 32 μg/ml plus 10^9^ PFU/ml; (E) ciprofloxacin, 16 μg/ml plus 10^8^ PFU/ml and 16 μg/ml plus 10^9^ PFU/ml; (F) ceftazidime, 2 μg/ml plus 10^4^ PFU/ml and 16 μg/ml plus 10^9^ PFU/ml; (G) chloramphenicol, 4 μg/ml plus 10^5^ PFU/ml and 4 μg/ml plus 10^9^ PFU/ml. Two-way ANOVA was employed for statistical significance testing. *, *P* < 0.05; **, *P* < 0.01; ***, *P* < 0.001; n.s., not significant. Growth curves show means ± standard deviations (SDs).

On the other hand, ExPEC harboring the enzyme CAT (JJ2528-CAT) was highly resistant to chloramphenicol; it took >32-fold more (1,024 μg/ml) chloramphenicol to achieve a similar reduction as the wild-type JJ2528 that lacks the enzyme (Fig. 1C, compare the left-most column to the same column in Fig. 1B). Once again, despite the presence of the CAT enzyme, combinatorial phage-antibiotic treatment resulted in a similarly high level of reduction of bacterial density as with low chloramphenicol dose, with a general downward trend in reduction observed to the lowest dose of chloramphenicol (0.5 μg/ml). As is true for the CAT-minus ExPEC, phage seemed to enhance chloramphenicol-based killing of the bacteria with little to no stimulation of the antibiotic on phage-based killing. In this regard, and as analyzed via the use of interaction plots (which allows one to determine if the effect is additive, synergistic, or antagonistic) (Fig. 2G), this type of phage-antibiotic killing can be described as an additive effect for both JJ2528 harboring either a chloramphenicol resistance gene or not. Interestingly, the interacting region for this synogram is highly dominated by phage killing with subtle variations in some wells. There were several combinations of lower titer of phage and higher doses of chloramphenicol in both wild-type JJ2528 and JJ2528-CAT that yielded statistically significant antagonistic interactions (Fig. 1B and C and 2G). Interactive regions of the synograms were analyzed by determining the area under the curve (AUC) and plotted as violin plots (see Fig. S1). These plots allow the visualization of the differences between synograms as a whole, and also they take into account how bacterial populations, subjected to different treatments, change over time. In general, the addition of a resistance gene shifted more of the combined treatment matrix to the right (a lower percent reduction) (Fig. S1A, green).

10.1128/mBio.01462-20.2 FIG S1 Violin plots for the interacting region of the synograms. Areas under the curves (AUCs) for the possible interacting regions of the synograms are shown in violin plots where the medians are the solid thick vertical lines and the quartiles are solid thin lines. (A) Comparison between bacterial resistance; (B) comparison between classes of antibiotics; (C) comparison between media. AUCs were normalized. Kruskal-Wallis test was performed, followed by Dunn’s test for multiple comparisons. *, *P* < 0.05; **, *P* < 0.01; ***, *P* < 0.001; ****, *P* < 0.0001. Download FIG S1, TIF file, 2.2 MB. Copyright © 2020 Gu Liu et al. 2020 Gu Liu et al. This content is distributed under the terms of the Creative Commons Attribution 4.0 International license.

The acquisition of a gene (such as that above) that encodes an enzyme or other protein that inactivates, blocks, or pumps out the antibiotic is one mode by which bacteria become resistant to antibiotics. Another mode, however, is that the gene acquires mutations that enhance the corresponding enzyme’s catalytic activity. To determine the effect of phage-antibiotic combinatorial treatment on bacteria harboring such changes, we introduced genes encoding the β-lactamases CTX-M-14 wild type (WT) and CTX-M-14 A77V/D240G into JJ2528. CTX is an enzyme that hydrolyzes the β-lactam ceftazidime; the presence of the double mutations (A77V/D240G) allows the bacteria to hydrolyze ceftazidime more efficiently than the wild-type enzyme (27). Mutant versions of CTX-M are correlated with clinically high rates of resistance and serve here as both a relevant and controlled model to determine the effect phage may have on treatment (28–30). In contrast to the previous introduction of CAT, which produced sharply defined resistance, the introduction of these β-lactamases yielded a subtle increase of resistance against ceftazidime, with an increase in the MIC of approximately 2-fold (Fig. 1D to F). In general, the ceftazidime synograms showed a single large interacting region. Many phage-antibiotic combinations efficiently killed planktonic cells, unlike the situation with chloramphenicol. In all three, a high degree of reduction was observed when phage was combined even with low antibiotic concentrations (0.5 μg/ml for JJ2528 WT, 0.5 μg/ml for JJ2528 CTX-M-14 WT, and 2 μg/ml for JJ2528 CTX-M-WT A77V/D240G), as opposed to that for ceftazidime-alone-treated cells (128 to 256 μg/ml for all three). Consistent with the synogram, there was more killing of JJ2528 WT (Fig. 1D) than of JJ2528 CTX-M-14 WT (Fig. 1E), while JJ2528 CTX-M-14 A77V/D240G showed the least reduction of bacteria at combinatorial concentrations (Fig. 1F; Fig. S1A, orange). However, the addition of the β-lactamase gene to wild-type cells had much less of a total effect on bacterial levels than the addition of chloramphenicol acetyltransferase (compare Fig. 1B and C to D and E). Unlike that observed for chloramphenicol, ceftazidime and ΦHP3 were synergistic over most of the concentrations of both agents, with as little as 1,000 PFU/ml reducing the MIC of the ceftazidime by 32-fold (Fig. 1D to F and Fig. 2F). This raises interesting questions as to how the specific mechanism of action of an antibiotic may also affect its ability to synergize with phage.

### Relationship between antibiotic mechanism of action and PAS.

**FIG 3:**
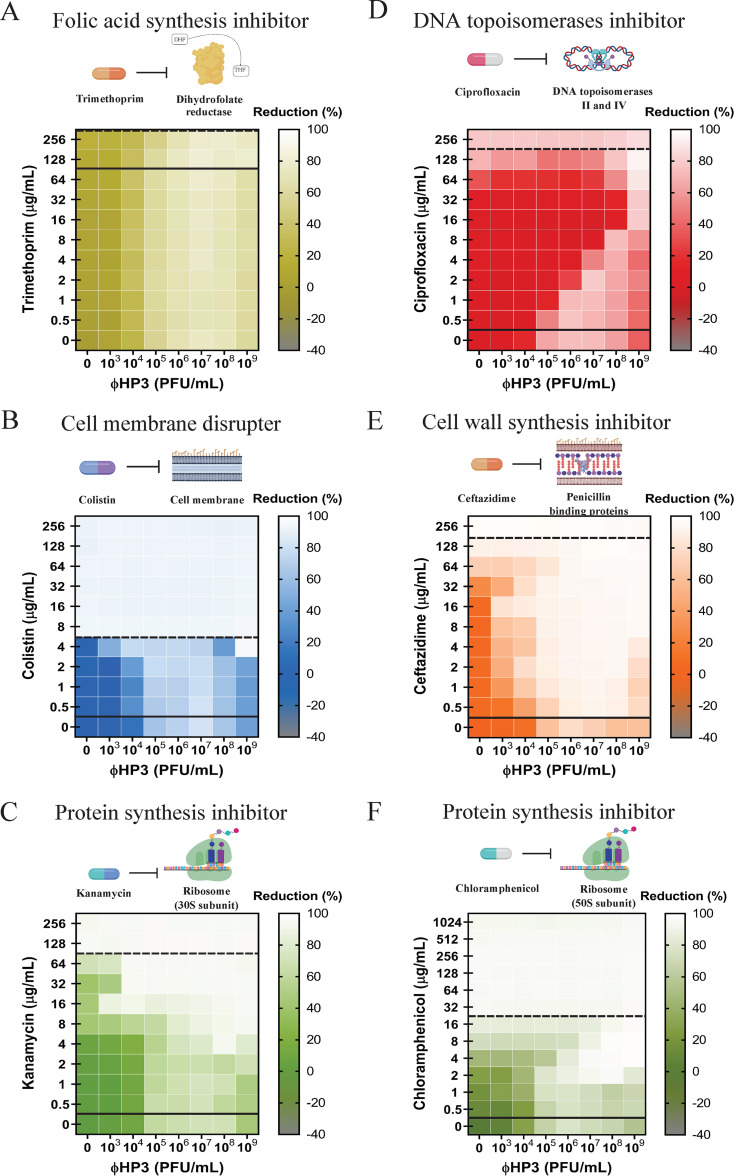
Effect of antibiotic class on phage-antibiotic synergy. A 100-fold diluted subculture of wild-type JJ2528 was incubated for 4 h, centrifuged, washed, adjusted too an OD_600_ of 1, and inoculated in a 96-well plate coated with ΦHP3 and antibiotics, and the OD_600_ was measured every 15 min for a total of 24 h with shaking. Effect of different antibiotics was studied with: (A) trimethoprim; (B) colistin; (C) kanamycin; (D) ciprofloxacin; (E) ceftazidime; (F) chloramphenicol. Synograms (*t* = 24 h) represent the mean reduction percentage of each treatment from three biological replicates: Reduction (%) = [(OD_growthcontrol_ − OD_treatment_)/OD_growthcontrol_] × 100. The regions above the dashed lines indicate antibiotic-mediated killing with highly effective doses; the regions between the solid and dashed lines represent the interacting regions of the phage and antibiotic, and the regions below the solid lines indicate phage-mediated killing with ineffective antibiotic concentrations.

The observation that two different classes of antibiotics (protein synthesis versus cell wall synthesis inhibitors) showed dramatically different interactions with the same phage over the same concentration of both agents suggested that the outcome of phage-antibiotic interactions might vary in a consistent manner by antibiotic class (or mechanism of action). To test this hypothesis, we determined the effect on JJ2528 when ΦHP3 was combined with representatives of four other classes of antibiotics, including trimethoprim (folic acid synthesis inhibitor), colistin (cell membrane disrupter), ciprofloxacin (DNA topoisomerases inhibitor), and kanamycin (protein synthesis inhibitor-30S subunit). Wild-type JJ2528 treated with ΦHP3 and the folic acid synthesis inhibitor trimethoprim produced a synogram dominated by phage killing (Fig. 3A). In fact, JJ2528 was not efficiently killed even on high trimethoprim concentrations (for example, 256 μg/ml). However, a reduction was achieved with either phage-alone-treated cells (≥10^4^ PFU/ml) or phage-antibiotic-treated cells (Fig. 3A). Of note, even the combined highest dose (256 μg/ml of trimethoprim and 10^7^ to 10^9^ PFU/ml of ΦHP3) was not statistically significant (Fig. 2B). This type of pairing can thus be classified as no effect (Fig. 2B, interaction plots).

The cell membrane disrupter, colistin, was very effective against wild-type JJ2528, in that even ≥8 μg/ml of colistin was able to lyse the cells almost completely (Fig. 3B). The combination of ΦHP3 and colistin produced mostly phage-dominated killing, where synergistic and antagonistic effects were only seen with one dose of 4 μg/ml and one phage titer of 10^4^ PFU/ml (Fig. 2C and 3B). In this case, unlike the cell wall biosynthesis inhibitor ceftazidime (synergism with phage) and the folic acid synthesis inhibitor trimethoprim (no effect), it seems the membrane disrupter colistin demonstrates both synergistic and antagonistic effects, similarly to that observed in the chloramphenicol synograms (protein synthesis inhibitor).

Finally, we also assessed the potential of ciprofloxacin, a DNA topoisomerase inhibitor, to act in combination with phage in killing JJ2528. Unexpectedly, the use of ΦHP3 and ciprofloxacin resulted in a highly patterned synogram (Fig. 3D) that resulted in a reduction of bacterial killing when the two agents were paired together (note the stepwise inhibition of bacterial killing as phage titers were increased). For instance, 8 μg/ml of ciprofloxacin combined with 10^7^ PFU/ml of ΦHP3 resulted in only 1% reduction, but the phage-alone treatment was around 73% reduction. This inhibition effect was then remediated by increasing the phage titer 10-fold (10^8^ PFU/ml), which led to 73% reduction. However, this seemingly effective combination was again reversed when the ciprofloxacin doubled to 16 μg/ml, which in combination with 10^8^ PFU/ml of ΦHP3, led to only 11% reduction (Fig. 3D). Thus, the use of ciprofloxacin resulted in two outcomes, antagonism and synergism (Fig. 2E), and a pattern that was not observed for any other antibiotic class to this point. This effect is also readily apparent when examining the AUC in a violin plot (Fig. S1B, red) compared to that for the other antibiotics.

### Assessment of two different antibiotics within the same mechanistic class.

We next determined how the synograms may change between two different drugs that act on the same cellular pathway, in this case, protein inhibition, but have subtle mechanistic differences in their action. For this, we chose to compare the 30S ribosomal subunit inhibitor kanamycin to the 50S ribosomal subunit inhibitor chloramphenicol. Interestingly, the synogram of kanamycin treatment closely resembled the synogram produced by JJ2528 CTX-M-14 A77V/D240G treated with ceftazidime and ΦHP3 (Fig. 1F and Fig. 3C). The kanamycin synogram also demonstrated increased combinatorial efficacy that resulted in effective killing compared to that with either treatment alone (Fig. 3C). For instance, more than 90% reduction was observed in the combination treatment, even with 4 μg/ml of kanamycin, whereas this degree of reduction in the kanamycin-alone-treated cells was only found at high doses (≥128 μg/ml). Moreover, JJ2528 WT seemed to have a subtle degree of resistance against kanamycin, just as JJ2528 CTX-M-14 A77V/D240G against ceftazidime. Through growth curves and interaction plots, kanamycin and ΦHP3 were determined to act synergistically with each other in some combinations, but additive in others (Fig. 2D). Note that chloramphenicol only produced additive effects with some subtle antagonistic interactions and that its synogram was very different than that observed for kanamycin. We also compared antibiotics of similar classes and/or mechanism of action. These include the cell wall synthesis inhibitors (ceftazidime versus cefepime), the DNA topoisomerase inhibitors (ciprofloxacin versus levofloxacin), the cell membrane disrupters (colistin versus polymyxin B), and folic acid synthesis inhibitors (trimethoprim versus sulfamethoxazole). In general, they exhibited similar effectiveness that was dependent on the class of antibiotic. However, there are variations between different antibiotics from the same mechanistic class, thus highlighting that there are other factors besides the known molecular mechanism of inhibition that may alter a given antibiotic’s ability to pair with a phage (see Fig. S4).

### Assessment of combinatorial treatment on preventing resistance.

**FIG 4:**
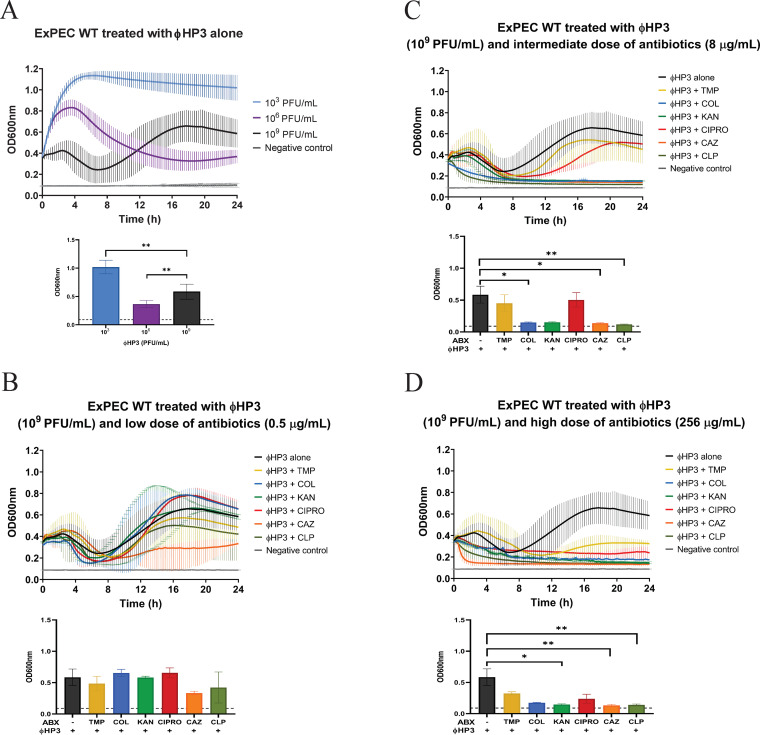
Effect of combinatorial treatment on preventing the rise of resistance. ExPEC strain JJ2528 wild-type cells were treated with different titers of ΦHP3-alone (A) and in combination with low (B), intermediate (C), and high (D) doses of antibiotics. Growth curves and bar graphs (*t* = 24 h) represent means ± SDs. Kruskal-Wallis test was performed, followed by Dunn’s test for multiple comparisons. *, *P* < 0.05; **, *P* < 0.01.

A phenomenon that was seen across some synograms is the revival of bacteria at 8 h when high titer of ΦHP3 (10^9^ PFU/ml) was applied as a single treatment (Fig. 4A) or as a combination with ineffective low dosage (0.5 μg/ml) of antibiotics (Fig. 4B). Interestingly, these “resistors,” if isolated and retested for sensitivity to phage ΦHP3, were completely recalcitrant to a second ΦHP3 challenge, which indicates they are true resistors (data not shown). The revival was prevented when ΦHP3 was applied along with an intermediate dose (8 μg/ml) of most antibiotics, except for trimethoprim and ciprofloxacin (Fig. 4C). JJ2528 treated with a high concentration (256 μg/ml) of trimethoprim and ΦHP3 still showed a second peak of growth, while high concentrations of the rest of the antibiotics combined with ΦHP3 prevented this revival (Fig. 4D). Of note, phage-alone-treated cells showed more fluctuations in bacterial levels, especially at later time points, than the positive control, antibiotic alone, and most dual-treated cells. This fluctuation was more evident at higher phage titers (both phage alone and combined) seen in multiple synograms.

### Assessment of host-like environments on phage-antibiotic efficacy.

**FIG 5:**
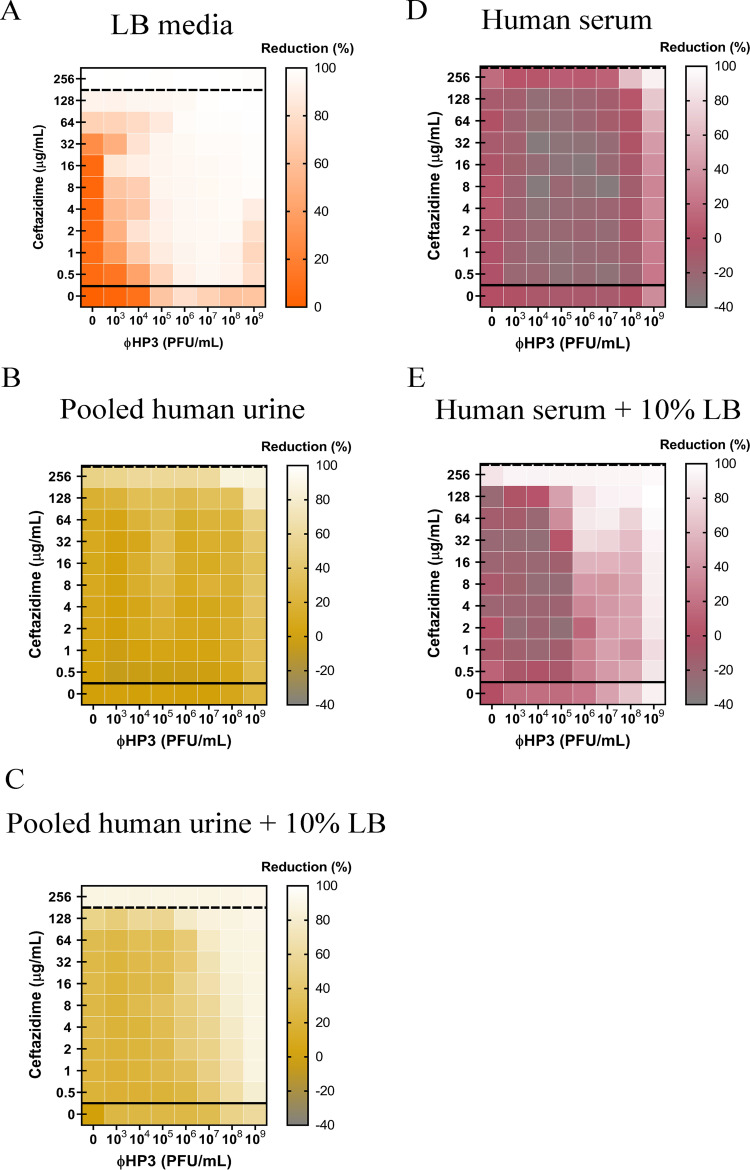
Effects of human urine and serum on phage-antibiotic synergy. A 100-fold diluted subculture of wild-type JJ2528 was incubated for 4 h, centrifuged, washed, adjusted to an OD_600_ of 1, and inoculated in a 96-well plate coated with ΦHP3 and ceftazidime. The OD_600_ was measured every 15 min for a total of 24 h for LB and urine and 8 h for serum with shaking. Bacterial cells were cultured in LB (A), pooled human urine (B), pooled human urine plus 10% LB (C), human serum (D), and human serum plus 10% LB (E). Synograms represent the mean reduction percentage for each treatment in urine (*N* = 3) and serum (*N* = 2): Reduction (%) = [(OD_growthcontrol_ − OD_treatment_)/OD_growthcontrol_] × 100. The regions above the dashed lines indicate antibiotic-mediated killing with highly effective doses; the regions between the solid and dashed lines represent the interacting regions of the phage and antibiotic, and the regions below the solid lines indicate phage-mediated killing with ineffective antibiotic concentrations.

The efficacy of any given antibiotic is not only influenced by the bacterium’s ability to inactivate or otherwise avoid its inhibitory effects but is also affected by the pharmacokinetics of antibiotics in a living organism. These include the antibiotic half-life in serum or tissues, whether the host modifies or inactivates the antibiotic, and its oral absorption or systemic dissemination when administered. These parameters are also expected to influence phage-antibiotic synergy as well as additional constraints the host places on phage, including, but not limited to, antagonism by the innate or adaptive immune response. To address the effect the host may have on PAS, we performed synography with two host physiologic environments important in ExPEC pathogenesis: blood and urine. In this context, blood is designed to simulate the behavior of bacteria and PAS under conditions that resemble systemic bacteremia, whereas urine is meant to simulate infections of the bladder. We used human pooled urine (from multiple donors to reduce variability) and human heat-inactivated serum (to eliminate any compromising negative effects on bacterial survival caused by complement). Note that even with additional heat inactivation, untreated bacterial levels decreased over time, especially >8 h; hence, we analyzed the serum synogram using the 8th hour as the endpoint. We first chose an antibiotic that synergizes well with ΦHP3 against wild-type JJ2528 in LB, which was the β-lactam ceftazidime (Fig. 5A). Interestingly, when this experiment was repeated with urine, the synogram was markedly different (Fig. 5B). The effective MIC of ceftazidime-alone-treated cells was raised to >256 μg/ml compared to that for the LB synogram. Despite being a completely different host environment with a different chemical composition, a similar effect was observed when the experiment was repeated with serum (Fig. 5D). Interactions were found in the upper right corner of each synogram, with high doses of each antimicrobial (for example, synergistic in urine and additive in serum). For the serum synogram, there was consistently antagonism in the entire range of interacting regions that did not display a pattern like that for the ciprofloxacin synogram in LB (Fig. 5D). Since untreated bacterial levels were less in urine and serum than in the nutrient-rich LB, we hypothesized that the overall reduction of killing in urine and serum was due to a lower growth rate of bacteria. Synography was performed again in both pooled human urine and human serum, but this time, LB was added to the urine and serum (final concentration, 10%). Adding LB to both urine and serum allowed synergism to appear at lower doses of dual-treated cells. Similarly, the serum plus 10% LB synogram seemed to have this trend; the antagonistic interactions in the combined treated cells appeared to have shifted toward the left side of the synogram as opposed to the serum-alone synogram (Fig. 5D and E).

### Assessment of the dependence of phage type on PAS.

**FIG 6:**
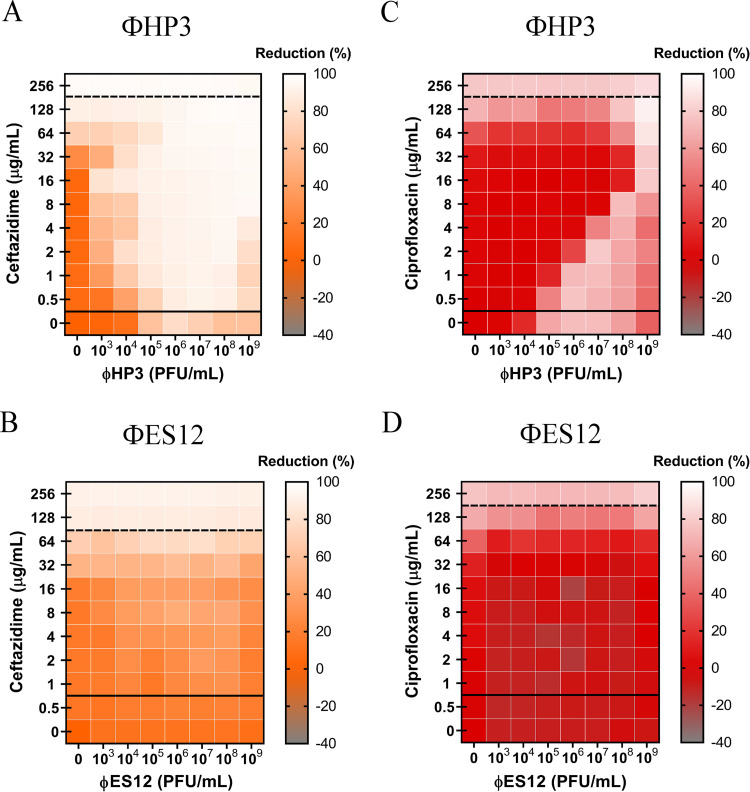
Effect of genetically similar phages on phage-antibiotic combined therapy. A 100-fold diluted subculture of wild-type JJ2528 was incubated for 4 h, centrifuged, washed, adjusted to an OD_600_ of 1, and inoculated in a 96-well plate coated with phages (ΦHP3 and ΦES12) and antibiotics in LB medium. OD_600_ was measured every 15 min for a total of 24 h with shaking in between. Synograms (*t* = 24 h) show wild-type ExPEC treated with ΦHP3 (A) and ΦES12 (B) with antibiotics (ceftazidime and ciprofloxacin). Synograms represent the average reduction percentage for each treatment from three biological replicates: Reduction (%) = [(OD_growthcontrol_ − OD_treatment_)/OD_growthcontrol_] × 100. The regions above the dashed lines indicate antibiotic-mediated killing with highly effective doses; the regions between the solid and dashed lines represent the interacting regions of the phage and antibiotic, and the regions below the solid lines indicate phage-mediated killing with ineffective antibiotic concentrations.

To determine if the types of PAS observed to this point are affected by the choice of phage, we chose to perform synography with phage that is 98% identical to ΦHP3 (termed ΦES12) but harbors a 6-fold reduced burst size (60 PFU/cell for ΦHP3 compared to 10 PFU/cell for ΦES12). We examined synography when ΦES12 was combined with ceftazidime (synergism previously observed with ΦHP3) and ciprofloxacin (antagonism previously observed with ΦHP3). Unexpectedly, the combination of ΦES12 and ceftazidime resulted in a synogram with mostly additive effects and a few synergistic combinations, with the overall patterning very different in composition (compare Fig. 6A with 6B). The combination of ΦES12 and ciprofloxacin yielded a synogram with some antagonistic interactions but also yielded a quite distinct pattern compared to that observed with ΦHP3 (Fig. 6C and D). In some respects, the latter synogram was more similar to the synogram observed when ΦHP3 was combined with ceftazidime and serum (Fig. 5D).

## DISCUSSION

Recognizing that a phage used in combination with antibiotics might yield possible beneficial interactions that can enhance *in vivo* efficacy of the antimicrobials, improve clinical outcomes, and decrease resistance, we tested a recently discussed phage highly specific against the pandemic ST131 clonal group of E. coli for synergistic interactions with all major classes of antibiotics. Primary findings from our study reveal (i) the development of a new high-throughput platform that quickly assesses the effect of various phage and antibiotic concentrations on bacterial growth, an analysis we call synography (and the resulting data represented in a synogram); (ii) that synograms demonstrate a wide range of conditions in which combinatorial treatment is synergistic, additive, or antagonistic, sometimes all three present in the same analysis; (iii) that a phage may demonstrate highly effective killing or inhibition when combined with one class of antibiotics but may lack this same effect when combined with another class; (iv) that phage may restore the competency of antibiotics even in bacteria that encode resistance elements against the chosen antibiotic, an effect we term “phage adjuvation” because the phage adjuvates, or make better, the antibiotic; (v) that highly genetically similar phages produce dramatically different synograms even when these are combined with the same class of antibiotics; (vi) that phage-antibiotic synergy may prevent resistance, but only when the antibiotic concentration is increased; and finally, (vii) the host-like conditions substantially influence PAS, and the synogram profiles in general, thus reflecting the need to test such antibacterial effects under conditions that more reliably simulate the host environment. In this case, the dampening of PAS seems to be due to a reduced growth rate when the bacterium is in urine or blood.

### Efficacy of combinatorial treatment in wild-type and antibiotic-resistant bacteria.

An area of phage therapy that is in its infancy is whether phage can help resensitize resistant bacteria toward antibiotics. Often, the options for antibiotics can be limited by bacterial resistance or by the patient’s own medical conditions. In this investigation, we studied the efficacy of combinatorial therapy using antibiotics that can be targeted by bacterial enzymes; these antibiotics are rendered ineffective if they were to be used alone. We found that the MIC was lowered for both wild-type and resistant JJ2528 when the antibiotic was combined with phage. In other words, phages seemed to act as a type of adjuvant to antibiotics, much like we consider alum for antigens in vaccines, even with bacteria harboring genetic elements that confer resistance to the antibiotic. In this sense, they “resensitize” bacteria by lowering the dose of antibiotic needed to achieve a similar reduction of bacterial levels compared to that for the antibiotic alone. There are other ways in which bacteria can be resensitized, for instance, through transduction whereby sensitizing genes are delivered to bacteria after phage infection (31). As noted here, the environmentally isolated phage in our study is already very efficacious in resensitizing a multidrug-resistant JJ2528 without prior transduction events. In this regard, it seems that such an effect is heavily dependent on finding the “optimal” dose of both agents such that their individual molecular mechanisms of action do not interfere with each other. In addition, synograms between resistant and wild-type JJ2528 showed similar types of interactions in general. For example, if synergism is found between a particular combination of phage and antibiotic in wild-type bacteria, just as the case of ΦHP3 and ceftazidime, this synergism also extends to resistant bacteria. The same applies to the additive-antagonistic effect found in both chloramphenicol-sensitive JJ2528-WT and chloramphenicol-resistant JJ2528-CAT. These results imply that the stoichiometry of each agent (e.g., the relative molar amounts of each) is an important determinant of activity and can be used to overcome the genotype of the pathogenic bacterium.

### The class of antibiotic determines the type of interaction with phage.

The possible types of interactions between phage and antibiotic were largely dictated by the class of antibiotics employed during therapy. In our system of wild-type JJ2528 treated with the same phage but with different antibiotics, we (i) observed additivism, synergism, antagonism, and neutrality; (ii) found that synergistic effects with an antibiotic do not translate to a different antibiotic of the same class that targets similar bacterial pathways; and (iii) showed that more than one type of interaction can exist simultaneously. Since there is a myriad of interactions between phage and antibiotic, there lies a possibility that the interference of some bacterial processes by certain antibiotics may also affect the lytic cycle of the phage. For instance, the synergism and antagonism seen in the membrane disrupter colistin can be caused either by the complexity of the drug (major and minor forms with amphiphilic property) or by its primary interaction with lipopolysaccharide (LPS) that leads to cell membrane destabilization (32, 33). Since many phages use LPS as a receptor on the bacterial cell, it is possible that the synogram reflected colistin affecting the initial adsorption stage of the phage (34, 35). Another example is the synergistic interaction found between ΦHP3 and the cell wall inhibitor ceftazidime, which can be correlated with the increased phage production caused by cephalosporins. These antibiotics have been demonstrated to increase cell filamentation as well as to cause the production of more phage particles (21, 36–38). It is also possible that the combined action of phage-derived products, which can disrupt the bacterial membrane integrity, along with the action of ceftazidime on the cell wall may cause a fragile barrier that allows easier cell lysis. The dose-dependent pattern between ciprofloxacin and ΦHP3 is a clear antagonistic behavior that can be explained by the primary targets, DNA topoisomerases, that are involved in DNA replication encoded by both bacterium and phage (39). ΦHP3, used in this study, encodes two subunits of DNA topoisomerase II (data not shown), suggesting that ciprofloxacin inhibits both the bacterial and the phage topoisomerases. Similarly, recent biofilm PAS studies noted that sequentially treating cells with phage and ciprofloxacin (noted synergism) instead of a simultaneous application (noted antagonism), may have allowed phage replication to occur first before ciprofloxacin’s interruption (23, 40). These results raise the possibility that the type of interactions in each phage-antibiotic combination is heavily dictated by the primary target of the antibiotic and the cellular processes required for phage replication (36, 41, 42). Lastly, since protein synthesis inhibitors most likely would interfere with phage production, the dominant synergistic effects seen with kanamycin were unexpected. Other PAS studies with protein synthesis inhibitors have also found synergistic interactions *in vitro* (41, 43). Thus, we speculate that ΦHP3 possesses a mechanism to bypass the antibiotic inhibition of the ribosome and allow synthesis of phage proteins. This notion is supported by the discovery of phage-encoded ribosomal subunits (44). The bactericidal property of kanamycin may have contributed to the enhanced killing found only in the kanamycin synogram. Bactericidal agents, such as kanamycin, accelerate cellular respiration rate, followed by stimulation of hydroxyl radicals that are thought to cause cell death (45). Since phage replication is thought to rely on metabolically active bacteria, increasing cellular respiration may enhance phage-mediated killing (46, 47).

### PAS prevents development of phage resistance.

From an evolutionary standpoint, the imposition of two different selective pressures on bacteria may reduce the chances for them to develop potential resistance (48). In our study, it was observed that combinatorial treatment prevented the rise of secondary bacterial growth that was often observed in phage-only-treated cells at later time points. This is consistent with studies that have demonstrated that only combinatorial therapy can effectively prevent the rise of phage-resistant variants (49–51). Such an observation bodes well for the prospects of using PAS to prevent resistance.

### The host physiological environment changes the efficacy of PAS.

Susceptibility testing involves tightly regulated parameters that include media, bacterial inoculum, dilutions of antibiotic, as well as the plate used for the assay (52). However, *in vitro* testing may not always correlate to *in vivo* efficacy. An inoculum effect was discovered to be an *in vivo* concern, and this represents only one of the tightly regulated parameters (53). Generally speaking, for all antibiotics tested here (except for trimethoprim, which was ineffective), as the concentration of bacteria increases, the effectiveness of the antibiotic decreases, consistent with the antibiotic being slowly dosed out (see Fig. S2 in the supplemental material). To study the potential impact that the physiological environment may have on phage-antibiotic interactions, medical simulators such as human urine and human serum were employed in place of the LB medium. Urine has been shown to increase the apparent MIC of E. coli to several classes of antibiotics (54), and this phenomenon was observed here. We also found that urine affected the efficacy of phages. Only the dual treatment showed an effective reduction of bacterial levels, and synergism was preserved, albeit modestly. This suggests that phage and ceftazidime are also effective in synergizing with each other even in acidic environments such as urine. Since phages are routinely used to treat urinary tract infections (UTI) at the Eliava Institute in Georgia, and there are increasing reports of successful phage therapy on UTI, more investigation is needed to understand the parameters required for successful treatment in the urine environment (55, 56). Phages face additional challenges in a complicated host system such as the blood that may lead to phage inactivation. In our serum synogram, there were antagonistic interactions observed only in combined treatment that can only be overcome by higher doses of ceftazidime and higher phage titers. It appears that serum does not cause ceftazidime to antagonize phage killing the way that ciprofloxacin does in LB. Conversely, the effect seems to be more of a failure to inactivate bacteria when both antimicrobials are present in serum, as opposed to single agents, and highly dependent on active bacterial growth. It would be of interest to screen for phages that somehow activate bacterial growth.

10.1128/mBio.01462-20.3 FIG S2 Inoculum effects of different antibiotics. A 100-fold diluted subculture of ExPEC (strain JJ2528) was incubated for 4 h, centrifuged, washed, adjusted to an OD_600_ of 1.2, serially diluted, and inoculated in a 96-well plate coated with different antibiotic concentrations. The lowest dose of antibiotic that inhibited visible bacterial growth at the end of 24 h was marked as the MIC for each bacterial inoculum. Inoculum effect was tested against different bacterial transformants with ceftazidime and chloramphenicol: (A) chloramphenicol on JJ2528-WT and JJ2528-CAT; (B) ceftazidime on wild-type JJ2528, JJ2528 CTX-M-14 wild-type, and JJ2528 CTX-M-14 A77V/D240G. Inoculum effect was also tested on JJ2528 wild-type with different antibiotics: (C) trimethoprim; (D) colistin; (E) kanamycin; (F) ciprofloxacin. Dashed lines represent the limits of detection. Download FIG S2, TIF file, 1.8 MB. Copyright © 2020 Gu Liu et al. 2020 Gu Liu et al. This content is distributed under the terms of the Creative Commons Attribution 4.0 International license.

### Similar, but distinct, phages result in different synograms.

Phages that are chosen for therapy should be carefully characterized to avoid the presence of potentially harmful genes that yield toxins, antibiotic resistance, and virulence to the bacterial host. From our recent characterized phage library (26), we chose to study antibiotic-phage interactions using ΦES12. Similar to ΦHP3, this phage is devoid of potentially harmful genes, and its genome sequence is 98% identical to that of ΦHP3. They have similar genome sizes (ΦHP3, 168 kb; ΦES12, 166 kb), G+C contents (ΦHP3, 35.4%; ΦES12, 35.37%), numbers of open reading frames (ΦHP3, 274; ΦES12, 267), and numbers of tRNAs (ΦHP3, 11; ΦES12, 9), similar absorption within 10 min (ΦHP3, 98%; ΦES12, 93%), and similar latent periods (ΦHP3, 22.5 min; ΦES12, 26 min), but they differ in burst size (ΦHP3, 60 PFU/ml; ΦES12, 9.6 PFU/ml) (26). Since the burst size is high in ΦHP3 and low in ΦES12, and burst size is one of the factors that affect phage-mediated killing, we hypothesize that the differences in the synograms observed between these phages might be due to this factor. However, other phage parameters, including adsorption rate and latent period, along with burst size, should undergo a comprehensive assessment as to how they affect synergy.

Overall, our investigation of phage-antibiotic interactions paves a new path to explore the complexity of success of dual therapy, especially under physiological conditions. The approach taken in this study can be used to inform clinicians on the possible antagonism that may arise during PAS testing. The real value of the findings reported here is they reveal that the phage-antibiotic interaction is quite complex and influenced by many factors that have not readily been systematically studied. Our work demonstrates (i) how different interactions between phage and antibiotic are strongly affected by the class of antibiotics, (ii) how phage generally lowers the MIC of the antibiotic, (iii) how phage and antibiotics suppress resistance, (iv) how bacterial resistance toward antibiotics impacts the combination therapy, (v) how host factors such as urine and serum affect these types of interactions, and (vi) how similar phages may result in dramatically different outcomes. For future studies, synograms can be modified to accommodate the combination of a phage cocktail combined to an antibiotic or a phage combined to multiple antibiotics. In addition, determining the efficacy of a phage cocktail combined with antibiotics in complex host systems, the effect of simultaneous versus sequential treatment to reduce antagonistic interactions, and the actual mechanisms behind each synergistic and antagonistic effect in combinatorial treatment are fertile grounds for future research.

## MATERIALS AND METHODS

### Bacterial culture, plasmids, phage, and antibiotics.

The clinical isolate ExPEC ST131 strain JJ2528 used in this study was kindly provided by James R. Johnson (57), and the plasmids (pTP123-CTX-M-14 WT and pTP123-CTX-M-14 A77V/D240G) that confer antibiotic resistance were kindly provided by Timothy Palzkill (27). The phages used in this study, ΦHP3 (accession number KY608967) and ΦES12 (accession number MN508614), were previously isolated from environmental sources (25, 26). All antibiotics were prepared fresh and filter sterilized (except chloramphenicol and trimethoprim due to solvent used for these antibiotics). Information about maintenance of bacterial culture, phage purification, classes of antibiotics, and solvent employed for antibiotics can be found in Text S1 in the supplemental material.

10.1128/mBio.01462-20.1 TEXT S1 Supplemental methods. Download Text S1, DOCX file, 0.01 MB. Copyright © 2020 Gu Liu et al. 2020 Gu Liu et al. This content is distributed under the terms of the Creative Commons Attribution 4.0 International license.

### Synergy testing in LB medium, human urine, and human serum.

Synergy testing was performed with LB medium, human pooled urine, and commercial human serum. A subculture of E. coli strain JJ2528 was incubated for 4 h in LB, centrifuged, washed, and recentrifuged. The pellet was resuspended in the medium under test and adjusted to ∼1 × 10^9^ CFU/ml (optical density at 600 nm [OD_600_] of 1), and 100 μl was inoculated into each well of the microtiter plate that contained the checkerboard of phage and antibiotic concentrations (50 μl for each antimicrobial). The OD_600_ was measured every 15 min at 37°C for a total of 24 h with continuous shaking in a Biotek Synergy HT (BioTek, Winooski, VT, USA). For more details on synergy assays, urine collection, and serum information, see Text S1.

### Inoculum effect.

To determine the effect of inoculum size on the efficacy of antibiotics, 2-fold serial dilutions of antibiotic were added to a 96-well plate. A bacterial subculture of JJ2528 was prepared as described for synergy testing, and serial 10-fold dilutions were inoculated into the microtiter plate and grown for 24 h at 37°C in a shaker. For each bacterial inoculum, the well with the lowest antibiotic concentration that showed bacterial clearance was marked as the MIC. For more details on antibiotics and bacterial dilution, see Text S1.

### Data representation and statistical analysis.

To generate synograms, absorbance readings from three biological replicates were normalized with the negative control, and the treated wells were deducted from the positive control (no treatment) to yield percent reduction: Reduction (%) = [(OD_growthcontrol_ − OD_treatment_)/OD_growthcontrol_] × 100.

Most of the synograms presented in this paper were generated using absorbance readings from *t* = 24 h. However, since datapoints were acquired every 15 min for a total of 24 h, synograms can be generated from multiple time points (see Fig. S3). Two-way analysis of variance (ANOVA) was employed on interaction plots to analyze possible synergism between phage and antibiotics. AUC was generated for the interacting region of each synogram and normalized. Some figures were generated with Biorender. For more details on the statistics, see Text S1.

10.1128/mBio.01462-20.4 FIG S3 Synograms of different time points for ceftazidime and ciprofloxacin. A 100-fold diluted subculture of wild-type JJ2528 was incubated for 4 h, centrifuged, washed, adjusted to an OD_600_ of 1, and inoculated in a 96-well plate coated with ΦHP3 and antibiotics, and the OD_600_ was measured every 15 min for a total of 24 h with shaking. Synograms were computed for different time points (8 h, 16 h, and 24 h) for the following antibiotics: (A) ceftazidime; (B) ciprofloxacin. Synograms represent the mean reduction percentage of each treatment from three biological replicates: Reduction (%) = [(OD_growthcontrol_ − OD_treatment_)/OD_growthcontrol_] × 100. The regions above the dashed lines indicate antibiotic-mediated killing with highly effective doses; the regions between the solid and dashed lines represent the interacting regions of the phage and antibiotic, and the regions below the solid lines indicate phage-mediated killing with ineffective antibiotic concentrations. Download FIG S3, TIF file, 1.1 MB. Copyright © 2020 Gu Liu et al. 2020 Gu Liu et al. This content is distributed under the terms of the Creative Commons Attribution 4.0 International license.

10.1128/mBio.01462-20.5 FIG S4 Synograms of antibiotics with similar mechanisms of action. A 100-fold diluted subculture of wild-type JJ2528 was incubated for 4 h, centrifuged, washed, adjusted to an OD_600_ of 1, and inoculated in a 96-well plate coated with ΦHP3 and antibiotics, and the OD_600_ was measured every 15 min for a total of 24 h with shaking. Synograms (*t* = 24 h) were computed for the following classes: (A) cell wall synthesis inhibitors (ceftazidime and cefepime); (B) DNA topoisomerases inhibitors (ciprofloxacin and levofloxacin); (C) cell membrane disrupters (polymyxin E and polymyxin B); (D) folic acid synthesis inhibitors (trimethoprim and sulfamethoxazole); (E) protein synthesis inhibitors (kanamycin and chloramphenicol). Synograms (*t* = 24 h) represent the mean reduction percentage of each treatment from at least two biological replicates: Reduction (%) = [(OD_growthcontrol_ − OD_treatment_)/OD_growthcontrol_] × 100. The regions above the dashed lines indicate antibiotic-mediated killing with highly effective doses; the regions between the solid and dashed lines represent the interacting regions of the phage and antibiotic, and the regions below the solid lines indicate phage-mediated killing with ineffective antibiotic concentrations. Download FIG S4, TIF file, 1.1 MB. Copyright © 2020 Gu Liu et al. 2020 Gu Liu et al. This content is distributed under the terms of the Creative Commons Attribution 4.0 International license.

10.1128/mBio.01462-20.6 FIG S5 Diagram of a 96-well plate showing raw absorbance readings for the combinatorial treatment on wild-type JJ2528. A 100-fold diluted subculture of ExPEC (strain JJ2528) was incubated for 4 h, centrifuged, washed, adjusted to an OD_600_ of 1.0, and inoculated in a 96-well plate coated with different antibiotic concentrations and phage titers. The plate shows the raw absorbance readings for one biological replicate. Download FIG S5, TIF file, 1.2 MB. Copyright © 2020 Gu Liu et al. 2020 Gu Liu et al. This content is distributed under the terms of the Creative Commons Attribution 4.0 International license.
