# Supplementary figures and images for: Phage-Antibiotic Synergy Is Driven by a Unique Combination of Antibacterial Mechanism of Action and Stoichiometry
Source: mBio. 2020 Aug 4;11(4):e01462-20. doi: 10.1128/mBio.01462-20 (PMC7407087; doi:10.1128/mBio.01462-20)

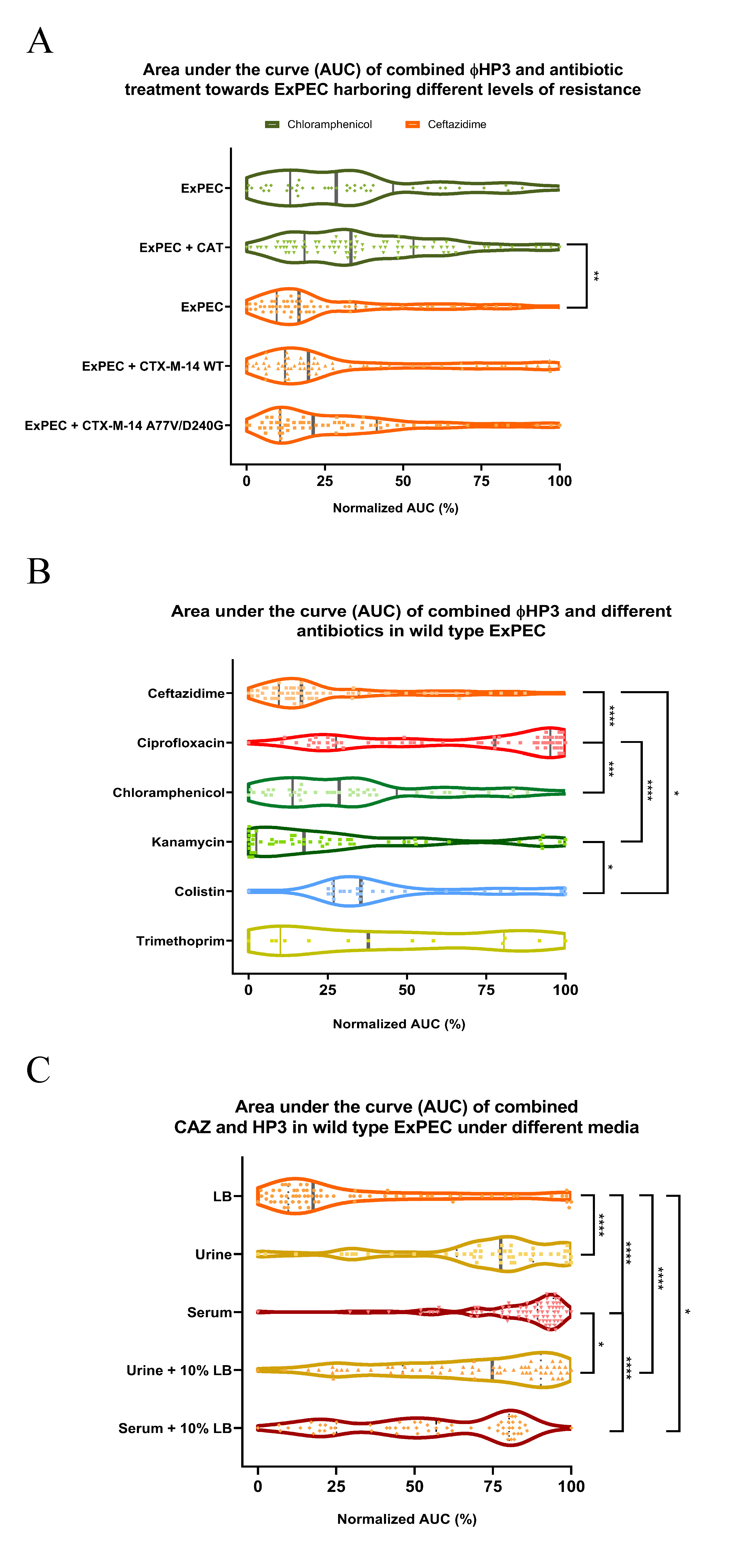

Supplement: FIG S1 [file mBio.01462-20-sf001.tif]

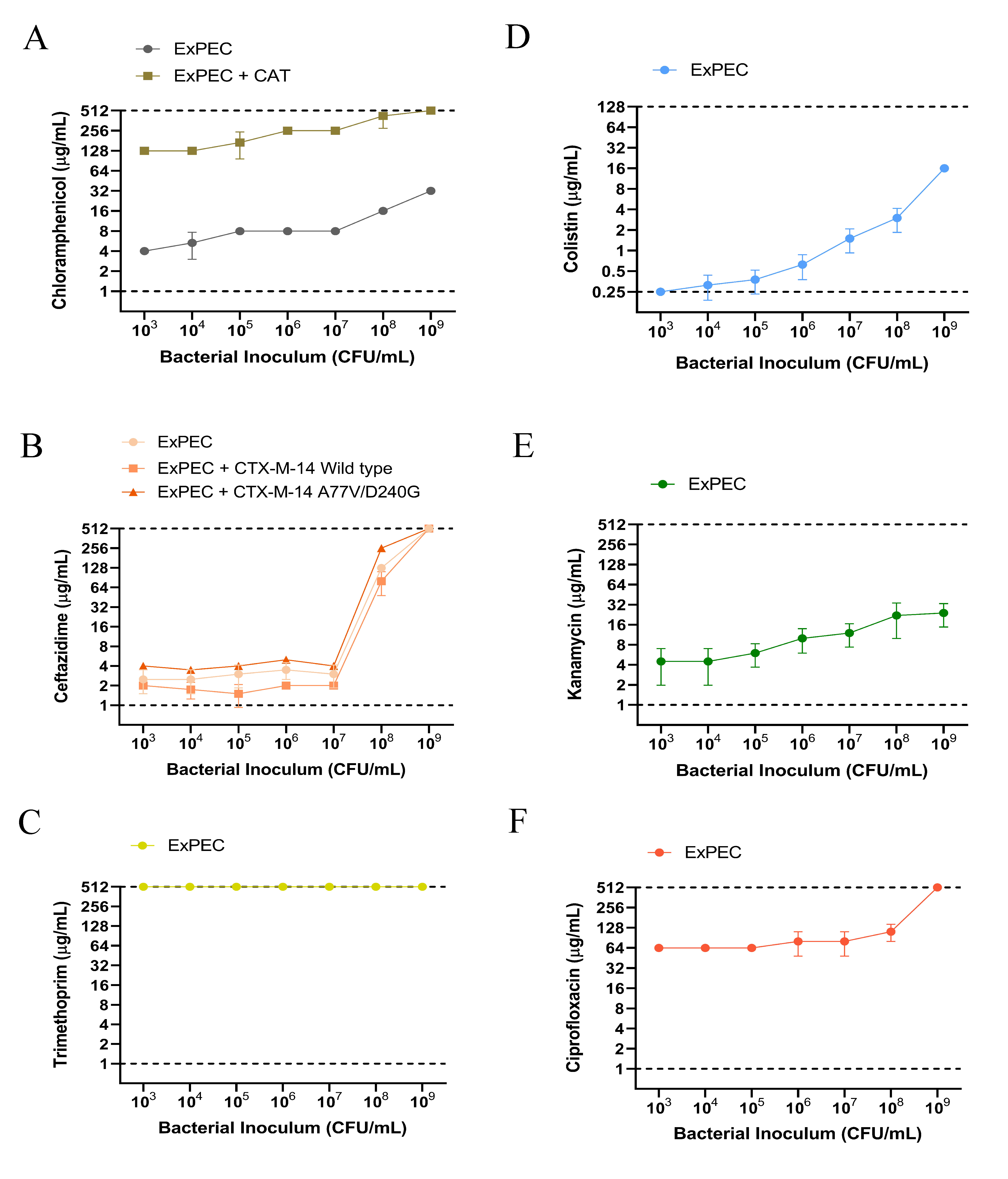

Supplement: FIG S2 [file mBio.01462-20-sf002.tif]

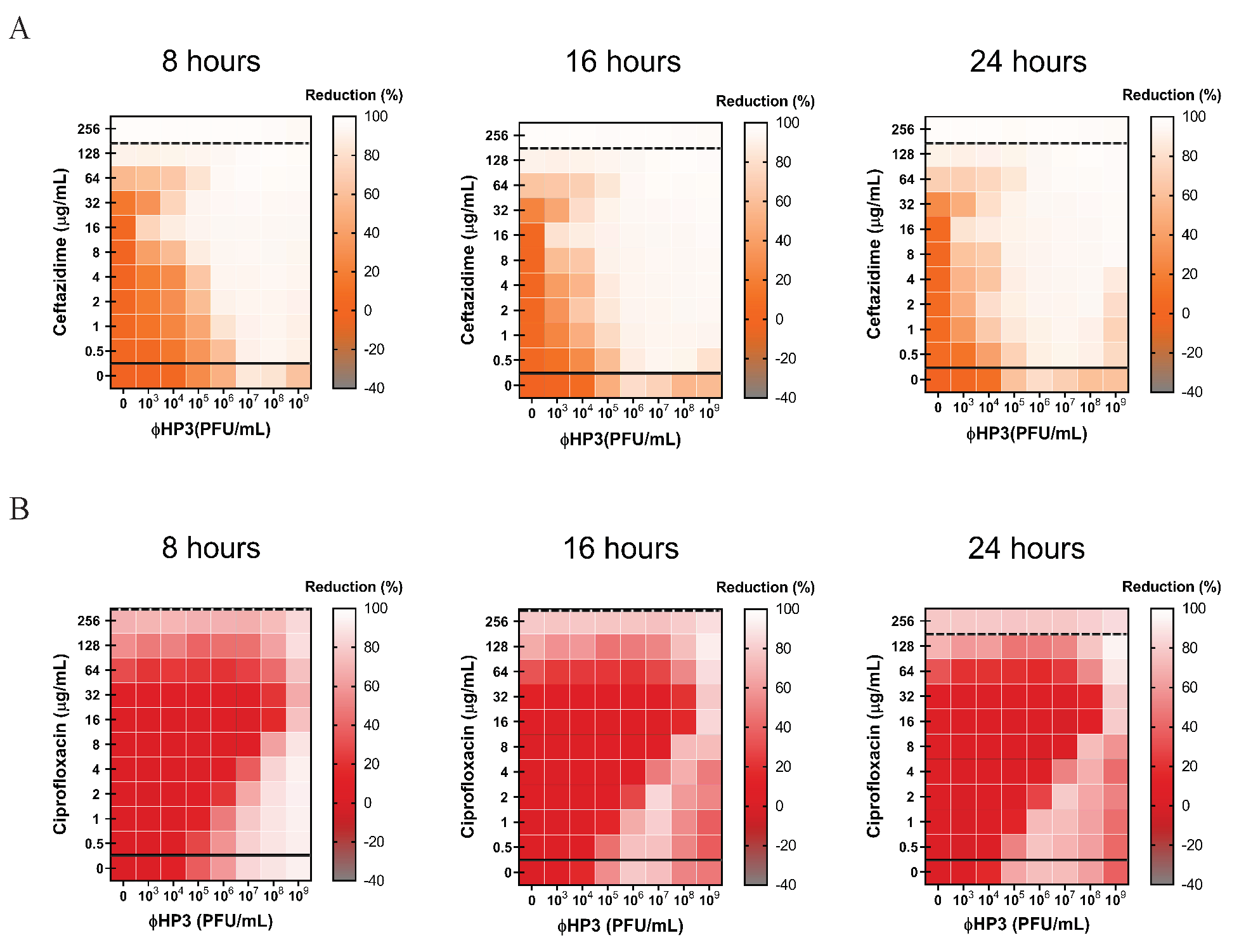

Supplement: FIG S3 [file mBio.01462-20-sf003.tif]

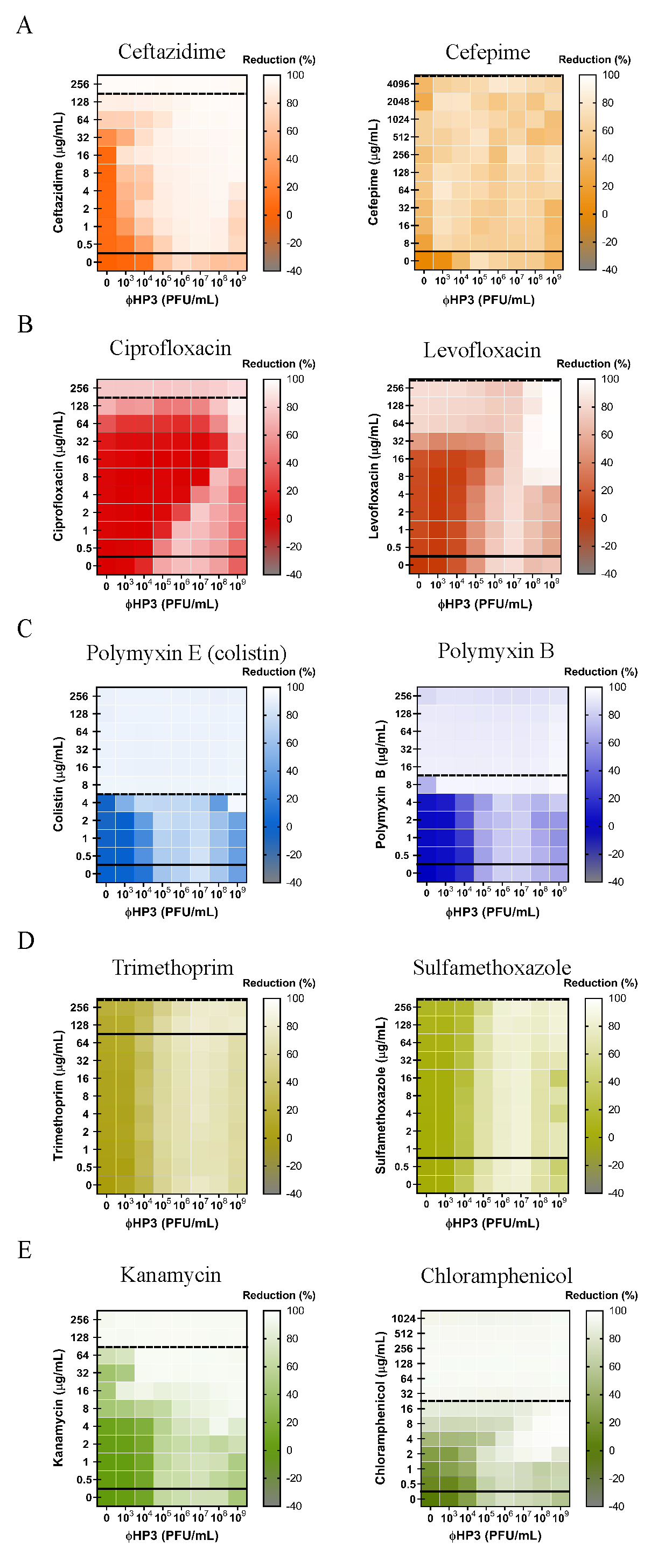

Supplement: FIG S4 [file mBio.01462-20-sf004.tif]

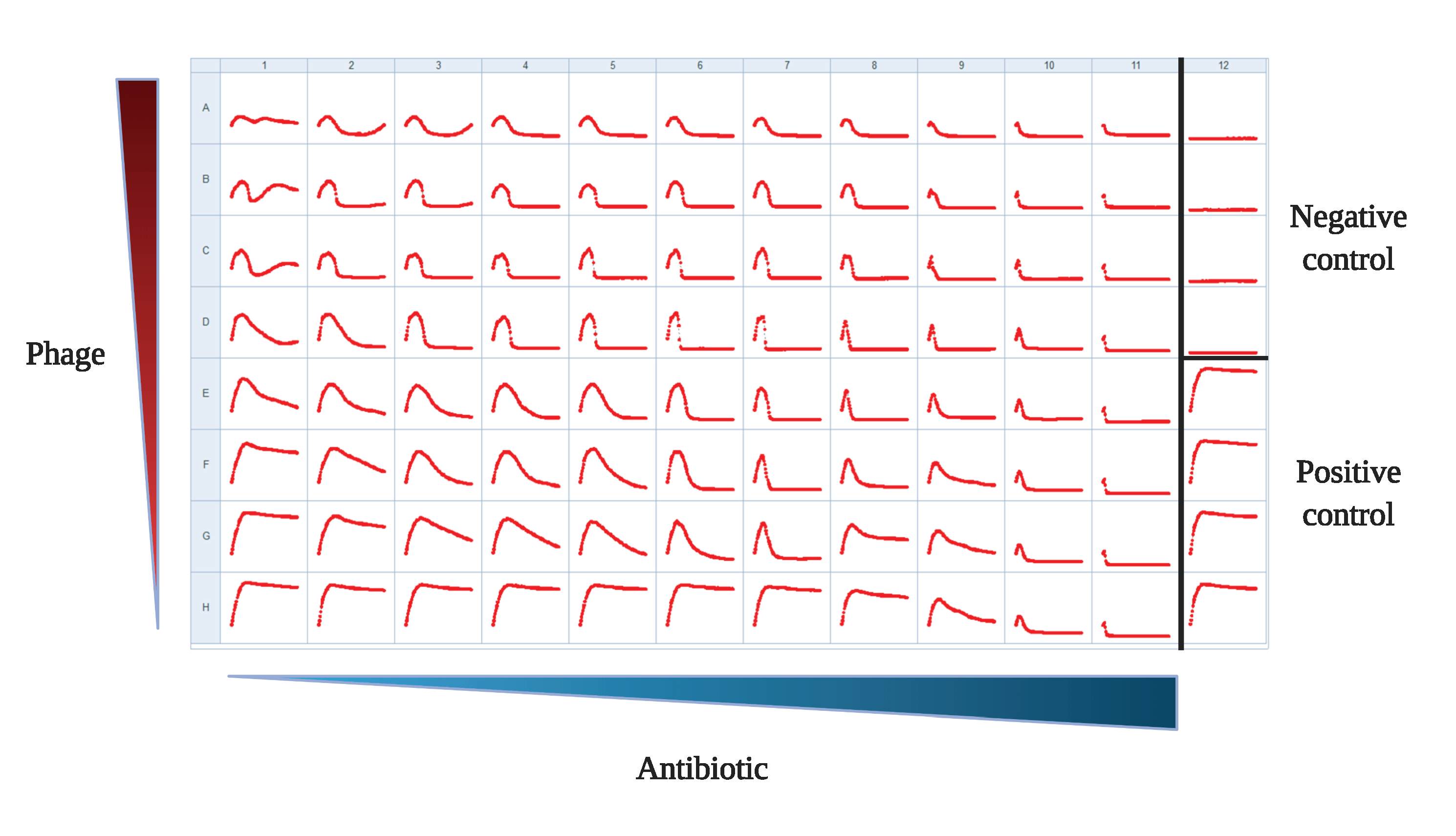

Supplement: FIG S5 [file mBio.01462-20-sf005.tif]
